# Multiphase modelling of the effect of fluid shear stress on cell yield and distribution in a hollow fibre membrane bioreactor

**DOI:** 10.1007/s10237-014-0611-7

**Published:** 2014-09-12

**Authors:** Natalie C. Pearson, Sarah L. Waters, James M. Oliver, Rebecca J. Shipley

**Affiliations:** 1 OCIAM, Mathematical Institute, University of Oxford, Andrew Wiles Building, Radcliffe Observatory Quarter, Woodstock Road, Oxford, OX2 6GG UK; 2 Biomechanical Engineering Group, Department of Mechanical Engineering, University College London, Torrington Place, London, WC1E 7JE UK

**Keywords:** Tissue engineering, Multiphase flow, Mechanotransduction, Asymptotic reduction

## Abstract

We present a simplified two-dimensional model of fluid flow, nutrient transport and cell distribution in a hollow fibre membrane bioreactor, with the aim of exploring how fluid flow can be used to control the distribution and yield of a cell population which is sensitive to both fluid shear stress and nutrient concentration. The cells are seeded in a scaffold in a layer on top of the hollow fibre, only partially occupying the extracapillary space. Above this layer is a region of free-flowing fluid which we refer to as the upper fluid layer. The flow in the lumen and upper fluid layer is described by the Stokes equations, whilst the flow in the porous fibre membrane is assumed to follow Darcy’s law. Porous mixture theory is used to model the dynamics of and interactions between the cells, scaffold and fluid in the cell–scaffold construct. The concentration of a limiting nutrient (e.g. oxygen) is governed by an advection–reaction–diffusion equation in each region. Through exploitation of the small aspect ratio of each region and asymptotic analysis, we derive a coupled system of partial differential equations for the cell volume fraction and nutrient concentration. We use this model to investigate the effect of mechanotransduction on the distribution and yield of the cell population, by considering cases in which cell proliferation is either enhanced or limited by fluid shear stress and by varying experimentally controllable parameters such as flow rate and cell–scaffold construct thickness.

## Introduction

The need for a reliable and sufficient source of replacement tissue and organs is constantly increasing, due to our ageing population and consistent lack of donors. The potential for tissue engineering to meet this demand is great, but there are still many barriers to be overcome before the field can provide alternative treatments on a clinical scale. For each tissue type, a different experimental technique must be developed to ensure that the engineered substitute is viable and can perform the same essential functions as the original tissue. This often means being able to mimic the in vivo environment of a particular tissue and has resulted in a vast array of protocols, bioreactors, culture conditions, and scaffold materials (see e.g. Martin et al. 2004; Martin and Vermette 2005; Pörtner et al. 2005; Stock and Vacanti 2001). For each set-up and cell type, optimal operating conditions must therefore be determined, a process which can be extremely time-consuming and expensive to resolve purely experimentally.

Mathematical modelling of bioreactor systems can be of great benefit for several reasons. It is reproducible and efficient, allowing the large numbers of parameters to be investigated relatively quickly and cheaply. Moreover, it can give insight into the combined effects of the physical processes involved in a particular set-up, or even focus on one process in isolation—this is hard to achieve in an experimental set-up. An example of one such process, and the focus of this work, is mechanotransduction: the mechanism by which forces are converted into biochemical signals and integrated into a cellular response. Below, we review a selection of mathematical models that specifically incorporate the effects of mechanotransduction. For more general detail on mathematical models in tissue engineering, we refer to the recent review by O’Dea et al. (2013a).

Previous work on multiphase modelling of mechanotransduction in tissue engineering includes a series of papers by O’Dea et al. (2008, 2010, 2013b). In O’Dea et al. (2008), the authors develop a two-phase model of tissue growth in a perfusion bioreactor. They include dependence of cell proliferation, extracellular matrix (ECM) deposition and cell death on the local cell density and fluid pressure. Results indicate that these effects can dramatically alter the composition of the resulting cell construct. This model was extended to include an additional porous scaffold phase in O’Dea et al. (2010), in which the system was simplified analytically by taking the long-wavelength limit appropriate for a small channel aspect ratio. The effect of fluid shear stress, as well as cell density and fluid pressure, was considered. Findings supported the earlier conclusion that mechanotransduction can significantly affect cell distribution. The same set-up was modelled and solved numerically by Osborne et al. (2010), highlighting situations in which a numerical approach is necessary, and when the analytical limits taken in O’Dea et al. (2010) are relevant. A further extension of this work is given in O’Dea et al. (2013b) in which the degradation of a scaffold is included, and whose spatially non-constant porosity is informed by experimental data.

As well as cell proliferation and death, mechanotransduction can affect cell differentiation. This is the focus of a paper by Byrne et al. (2007), in which finite element analysis is used to model a poroelastic tissue–scaffold construct for bone regeneration, with the objective of determining optimal scaffold porosity under different mechanical loading conditions. The dependence of stem cell differentiation on fluid velocity and shear strain is considered by employing the mechanoregulation algorithm developed by Prendergast et al. (1997). Depending on the stimulus level, differentiation into fibroblasts, chondrocytes or osteoblasts can occur. Aiming to identify possible treatment options in fracture healing, Lacroix and Prendergast (2002) develop a three-dimensional model of a human tibia fracture, again using the mechanoregulation algorithm proposed in Prendergast et al. (1997). Two different compressive loading magnitudes were simulated, and it was shown that only the lower load led to successful healing.

A finite element approach was also taken by Driessen et al. (2003), who modelled the effect of the different mechanical loading regimes resulting from closed and open configurations on collagen fibre content and orientation in an aortic valve. The mechanical properties of the tissue construct were shown to depend on the type of loading, as the fibres aligned with the principal strain directions, and the results were validated against experimental data where possible. Cardiac tissue was also the focus of the work in Latimer et al. (2003), who determined the stress and strain distribution in two- and three-dimensional tissues. Analytical solutions were found and used to demonstrate the strain in and around an ischemic region, and have the potential to be further used to predict mechanotransduction effects on the tissue leading to arrhythmias, or following hypoxia. Finally, the importance of scaffold design in bone tissue engineering motivated the study by Sanz-Herrera et al. (2009). The need for an implantable construct that has the required macroscale properties, but which is affected by biophysical phenomena on the microscale, led to a multiscale model which incorporated a realistic scaffold microstructure. Bone regeneration was assumed to be dependent on mechanical stimuli, and properties for the macroscale model such as the scaffold stress and strain and cell diffusion were obtained through the analysis of the underlying microstructure, which in turn evolved as a result of macroscale cell migration.

**Fig. 1:**
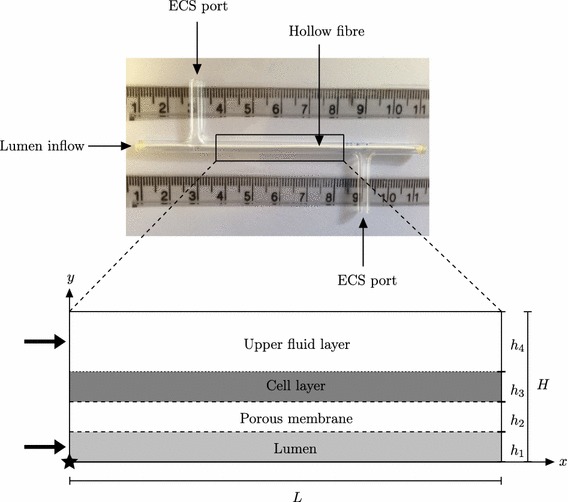
*Top* Photograph of a single HFMB module (ruler scale in cm) as shown in Pearson et al. (2013). *Bottom* cross section of the boxed region (not to scale), where the lower half (not shown) has been excluded based on symmetry. This depicts the idealised two-dimensional modelling region with the $x$ axis running along the lumen centreline. The *solid black arrows* show the direction and location of the fluid fluxes into the system, and the *star* denotes the origin $(x,y)=(0,0)$

In this paper, we consider various experimentally relevant case studies, motivated by specific cell types, to investigate the effect of fluid shear stress on the proliferation rate, and thus distribution, of cells in a hollow fibre membrane bioreactor (HFMB) (see Fig. 1). A HFMB consists of a cylindrical, glass module with a port at each end of the extracapillary space (ECS) and a porous hollow fibre inserted through the centre. A number of different cell seeding and flow regimes can be employed; for this work, we consider the set-up in which the cells are seeded in a scaffold in the ECS between the hollow fibre and the outer glass wall. Culture medium is pumped in via the lumen inlet and upstream ECS port. The pressure is set at the downstream lumen end via a clamp, and the downstream ECS port is left open to the atmosphere. Depending on the flow and pressure conditions, the fluid may pass through the hollow fibre walls and then flow out of either the lumen outlet or downstream ECS port.

We build on the model developed in Pearson et al. (2013) which describes fluid flow, solute concentration and cell distribution in a HFMB. In Pearson et al. (2013), the entire ECS was filled by the cell–scaffold construct, through which media could flow. Fluid velocities in the membrane and ECS were assumed to be an order of $\varepsilon$ smaller than those in the lumen as a result of the resistance to flow through these porous regions, where $\varepsilon \ll 1$ is the lumen aspect ratio. Here, we consider the alternative experimental set-up in which the cell–scaffold construct only fills part of the ECS (that directly above the porous membrane, defined as the cell layer), and media can flow freely throughout the remainder. The width of this cell layer can be controlled experimentally and will be varied as part of our investigation. Fluid is pumped into the system via both the lumen inlet and upstream ECS port; as a result, we expect the fluid velocity in this upper fluid layer to be of the same order of magnitude as the lumen. As well as enabling the relevant solutes to be delivered to, or removed from, the cells more quickly, we expect the cells to be exposed to a higher and potentially significant level of fluid shear stress as the flow through the cell layer will be enhanced by the upper fluid flow above. The impact of this set-up remains an open question experimentally: depending on the cell type, fluid shear stress can either be beneficial or detrimental to cell growth and survival. In this work, we therefore consider mechanotransduction effects, which were not included in Pearson et al. (2013). Specifically, we explore the effect of fluid shear stress on cell yield and distribution, via changes in the fluid flux into the upper fluid layer. In addition, the fluid flux affects the concentration of a nutrient (taken to be oxygen) which can limit the cell proliferation rate. We determine the range of possible behaviours from this set-up and find optimal flow rates for which the fluid shear stress has an advantageous effect: that is, it enables a more spatially uniform cell population and/or a higher cell yield to be obtained.

### Paper outline

We begin in Sect. 2 by describing the simplified modelling domain before introducing the governing equations and boundary conditions for each region in Sects. 2.1 and 2.2. In Sect. 3.1, we introduce relevant parameter values, motivating the non-dimensionalisation of the system (details of which are given in “Appendix”). In Sects. 3.2–3.4, we exploit the small aspect ratio of the lumen to reduce the system to two coupled partial differential equations for the cell volume fraction and solute concentration. Results for the cases in which cell proliferation is either enhanced or limited by fluid shear stress are presented in Sects. 4.1 and 4.2, and the sensitivity of our results to the cell layer width is investigated in Sect. 4.3. Finally, key findings and conclusions are discussed in Sect. 5.

## Model description

We describe the system using two-dimensional Cartesian coordinates, for simplicity and to enable analytical progress. Although results would be quantitatively different in an axisymmetric or three-dimensional set-up, we would not expect them to change qualitatively and hope to verify this in future work. We define the axial dimension of the modelling domain by $0<x<L$. The lumen and membrane are then respectively given by $0<y<h_1$ and $h_1<y<h_1+h_2$ in the transverse direction. The cell layer is of comparable thickness to the membrane, occupying $h_1 + h_2 < y < h_1 + h_2 + h_3$, and the upper fluid occupies $H-h_4<y<H$, where $H=h_1+h_2+h_3+h_4$ (see bottom half of Fig. 1). As in Pearson et al. (2013), we consider the cell layer to be a multiphase region consisting of the cells, culture medium (modelled as water) and a rigid, inert scaffold, closely following the formulation from Lemon et al. (2006). Water variables in the lumen, membrane, cell layer and upper fluid layer are denoted by subscripts $\mathrm {l},\ \mathrm {m},\ \mathrm {w}$ and $\mathrm {f}$, respectively, and cell phase variables by subscript $\mathrm {n}$, with the velocities given by $\mathbf {u}_i = (u_i,v_i)$, the water pressures by $p_i$, and the solute concentrations per unit volume of water by $c_i (i=\mathrm {l},\mathrm {m},\mathrm {w},\mathrm {f})$. In the cell layer, we track the dynamics of the cell, water and scaffold phases through their respective volume fractions $\theta _\mathrm {n}, \theta _\mathrm {w}$ and $\theta _\mathrm {s}$, where $\theta _\mathrm {s}$ is constant in space and time due to the assumption of a rigid, inert scaffold.

### Governing equations

The dimensional governing equations in each region take the form of conservation of mass and momentum for the water and cell phases, and conservation of mass for the solute. In the lumen, porous membrane and cell layer are the same as for the three-region system in Pearson et al. (2013), and the dynamics in the additional upper fluid layer are governed by the same equations as the lumen. We take the membrane porosity $\phi$ to be constant and work with reduced pressures throughout since gravitational effects are not negligible at the flow rates considered (see Pearson et al. 2013 for details). In the lumen ($0<y<h_1$), the relevant water equations are those of Stokes flow, along with an advection–diffusion equation for the solute:

2.1 $$\begin{aligned}&\nabla \cdot \mathbf {u}_\mathrm {l} = 0,\quad -\nabla p_\mathrm {l} + \mu _\mathrm {w}\nabla ^2\mathbf {u}_\mathrm {l} =\mathbf {0},\nonumber \\&\quad \frac{\partial c_\mathrm {l}}{\partial t} + \nabla \cdot (c_\mathrm {l}\mathbf {u}_\mathrm {l}) = D\nabla ^2 c_\mathrm {l}, \end{aligned}$$

where $t$ represents time, $\mu _\mathrm {w}$ the water viscosity (assumed constant) and $D$ the diffusion coefficient for the solute in water (also assumed constant). In the porous membrane ($h_1<y<h_1+h_2$), we use Darcy’s law for flow in porous media,

2.2 $$\begin{aligned}&\mathbf {u}_\mathrm {m} = -\frac{k}{\mu _\mathrm {w}}\nabla p_\mathrm {m},\quad \nabla \cdot \mathbf {u}_\mathrm {m} =0,\nonumber \\&\quad \frac{\partial (\phi c_\mathrm {m})}{\partial t} + \nabla \cdot (\phi c_\mathrm {m}\mathbf {u}_\mathrm {m}) = D\phi \nabla ^2 c_\mathrm {m}, \end{aligned}$$

where $k$ is the (constant) membrane permeability. In the cell layer ($h_1+h_2<y<H-h_4$), the no-voids condition is given by

2.3 $$\begin{aligned} \theta _\mathrm {n} + \theta _\mathrm {w} + \theta _\mathrm {s} = 1, \end{aligned}$$

while conservation of mass and momentum for the cell phase is given by

2.4 $$\begin{aligned} \frac{\partial \theta _\mathrm {n}}{\partial t}+\nabla \cdot (\theta _\mathrm {n}\mathbf {u}_\mathrm {n})&= J_\mathrm {n}, -\nabla (\theta _\mathrm {n}p_\mathrm {n})+\nabla \cdot (\theta _\mathrm {n}\varvec{\tau }_\mathrm {n})\nonumber \\&\quad + \psi _\mathrm {ns}\theta _\mathrm {s}\nabla \theta _\mathrm {n} - \gamma _\mathrm {ns}\theta _\mathrm {n}\theta _\mathrm {s}\mathbf {u}_\mathrm {n} = \mathbf {0}. \end{aligned}$$

Here, $J_\mathrm {n}$ represents the net cell production rate, $p_\mathrm {n}$ is the (reduced) cell pressure, $\psi _\mathrm {ns}$ is the interphase pressure due to tractions between the cells and scaffold, $\gamma _\mathrm {ns}$ is the (constant) cell–scaffold drag coefficient, and $\tau _\mathrm {n}$ is the deviatoric stress tensor for the cell phase, given by

2.5 $$\begin{aligned} \varvec{\tau }_\mathrm {n} = \mu _\mathrm {n}\left( \nabla \mathbf {u}_\mathrm {n} +\left( \nabla \mathbf {u}_\mathrm {n}\right) ^\mathrm {T} -\frac{2}{3}\left( \nabla \cdot \mathbf {u}_\mathrm {n}\right) \mathbf {I}\right) , \end{aligned}$$

where superscript $\mathrm {T}$ denotes transpose, $\mathbf {I}$ is the identity tensor and $\mu _\mathrm {n}$ is the effective viscosity of the cell phase (assumed to be much greater than $\mu _\mathrm {w}$). Here, we have neglected tractions and drag between the cells and water, assuming that they are much smaller than those between the cells and scaffold. This simplifying assumption is motivated by the expectation that the cells will strongly adhere to the scaffold. In the equations for the water phase, below we similarly neglect tractions between the cells and water. The corresponding equations for the water phase are

2.6 $$\begin{aligned}&\frac{\partial \theta _\mathrm {w}}{\partial t}+\nabla \cdot (\theta _\mathrm {w}\mathbf {u}_\mathrm {w}) = -J_\mathrm {n},\nonumber \\&\quad -\theta _\mathrm {w}\nabla p_\mathrm {w}-\gamma _\mathrm {ws}\theta _\mathrm {w} \theta _\mathrm {s}\mathbf {u}_\mathrm {w} = \mathbf {0}, \end{aligned}$$

where we have assumed that the net water production rate is $-J_\mathrm {n}$ so that mass is conserved. Conservation of solute mass in this region yields the following:

2.7 $$\begin{aligned} \frac{\partial (\theta _\mathrm {w}c_\mathrm {w})}{\partial t}+\nabla \cdot (\theta _\mathrm {w} c_\mathrm {w} \mathbf {u}_\mathrm {w})=D\nabla \cdot (\theta _\mathrm {w} \nabla c_\mathrm {w}) +\mathcal {R}, \end{aligned}$$

where $\mathcal {R}$ is a reaction term which accounts for solute uptake by the cells. Finally, in the upper fluid layer ($H-h_4<y<H$), the governing equations are

2.8 $$\begin{aligned}&\nabla \cdot \mathbf {u}_\mathrm {f} = 0,\quad -\nabla p_\mathrm {f} + \mu _\mathrm {w}\nabla ^2\mathbf {u}_\mathrm {f} = \mathbf {0},\nonumber \\&\quad \frac{\partial c_\mathrm {f}}{\partial t} + \nabla \cdot (c_\mathrm {f}\mathbf {u}_\mathrm {f}) = D\nabla ^2 c_\mathrm {f}. \end{aligned}$$

Constitutive forms need to be prescribed for a number of the terms above, namely $J_\mathrm {n}, \mathcal {R}$ (which we present and discuss in Sect. 4) and $p_\mathrm {n}$ and $\psi _\mathrm {ns}$. For now, we assume only that $J_\mathrm {n}$ and $\mathcal {R}$ are functions of the cell volume fraction $\theta _\mathrm {n}$, cell layer solute concentration $c_\mathrm {w}$ and (in the case of $J_\mathrm {n}$) the fluid shear stress in the cell layer. As will be seen in Sect. 3.2, the fluid flow in the cell layer is independent of $y$, and thus, we take the fluid shear stress to be proportional to the fluid pressure gradient $\partial p_\mathrm {w}/\partial x$. The cell pressure is assumed to be equal to the water pressure plus an extra component $\varPi$ which accounts for cell–cell interactions,

2.9 $$\begin{aligned} p_\mathrm {n} = p_\mathrm {w} + \varPi (\theta _\mathrm {n}), \end{aligned}$$

where

2.10 $$\begin{aligned} \varPi (\theta _\mathrm {n}) = \theta _\mathrm {n}\left( -\nu +\frac{\delta \theta _\mathrm {n}}{1-\theta _\mathrm {s}-\theta _\mathrm {n}}\right) , \end{aligned}$$

for constants $\nu$ and $\delta$. The form for the extra component $\varPi$ is motivated by O’Dea et al. (2010), and the first term models aggregation of cells at low densities, whilst the second term represents contact inhibition. As in Pearson et al. (2013), $\psi _\mathrm {ns}$ is assumed to be a negative constant representing the cells’ affinity for the scaffold:

2.11 $$\begin{aligned} \psi _\mathrm {ns} = -\eta . \end{aligned}$$

### Boundary conditions

On $y=0$ symmetry requires that

2.12 $$\begin{aligned} \frac{\partial u_\mathrm {l}}{\partial y} = 0,\quad v_\mathrm {l} = 0,\quad \frac{\partial c_\mathrm {l}}{\partial y} = 0; \end{aligned}$$

on the lumen/membrane interface $y=h_1$ we impose no slip of fluid, and continuity of fluid flux, of normal stress, of solute concentration and of solute flux, viz.

2.13 $$\begin{aligned} \begin{aligned}&u_\mathrm {l} = 0,\quad v_\mathrm {l} = \phi v_\mathrm {m},\\&\mathbf {n}_\mathrm {l}\cdot \varvec{\sigma }_\mathrm {l}\cdot \mathbf {n}_\mathrm {l} =\mathbf {n}_\mathrm {l}\cdot \varvec{\sigma }_\mathrm {m}\cdot \mathbf {n}_\mathrm {l},\\&c_\mathrm {l} = c_\mathrm {m},\quad \frac{\partial c_\mathrm {l}}{\partial y} = \phi \frac{\partial c_\mathrm {m}}{\partial y}; \end{aligned} \end{aligned}$$

and on the membrane/cell layer interface $y=h_1+h_2$ we impose no flux and no slip of cells, and continuity of fluid flux, of normal stress, of solute concentration and of solute flux, viz.

2.14 $$\begin{aligned} \begin{aligned}&\mathbf {u}_\mathrm {n} = \mathbf {0},\quad \phi v_\mathrm {m} = \theta _\mathrm {w}v_\mathrm {w},\\&\mathbf {n}_\mathrm {m}\cdot \varvec{\sigma }_\mathrm {m}\cdot \mathbf {n}_\mathrm {m} =\mathbf {n}_\mathrm {m}\cdot \varvec{\sigma }_\mathrm {w}\cdot \mathbf {n}_\mathrm {m},\\&c_\mathrm {m} = c_\mathrm {w},\quad \phi \frac{\partial c_\mathrm {m}}{\partial y} = \theta _\mathrm {w}\frac{\partial c_\mathrm {w}}{\partial y}. \end{aligned} \end{aligned}$$

On the cell layer/upper fluid interface $y=H-h_4$, we impose no flux and no shear stress on the cell phase, continuity of flux and of normal stress on the water phase, no slip of fluid and continuity of concentration and of flux of solute:

2.15 $$\begin{aligned} \begin{aligned} v_\mathrm {n} =&0,\quad \mathbf {n}_\mathrm {e}\cdot \varvec{\sigma }_\mathrm {n}\cdot \mathbf {t}_\mathrm {e}= 0,\\ u_\mathrm {f} =&0,\quad \theta _\mathrm {w}v_\mathrm {w} = v_\mathrm {f},\quad \mathbf {n}_\mathrm {e} \cdot \varvec{\sigma }_\mathrm {w}\cdot \mathbf {n}_\mathrm {e} = \mathbf {n}_\mathrm {e} \cdot \varvec{\sigma }_\mathrm {f}\cdot \mathbf {n}_\mathrm {e},\\ c_\mathrm {w} =&c_\mathrm {f},\quad \theta _\mathrm {w}\frac{\partial c_\mathrm {w}}{\partial y} = \frac{\partial c_\mathrm {f}}{\partial y}. \end{aligned} \end{aligned}$$

We note that, in the above, the most appropriate choice of stress condition on the cell phase is unclear, and we have chosen to apply no shear stress as an example first case. Equally, a nonzero shear stress condition could be chosen, and the analysis follows through in a similar manner. However, this would require prescription of another constitutive term for the partition of the shear stress, and hence, we have chosen not to consider this here for simplicity. Finally, on the top of the bioreactor $y=H$, we impose no slip and no flux of fluid, and no flux of solute:

2.16 $$\begin{aligned} \mathbf {u}_\mathrm {f} = \mathbf {0},\quad \frac{\partial c_\mathrm {f}}{\partial y} = 0. \end{aligned}$$

We impose boundary conditions at the up- and down-stream ends of the bioreactor once the system has been reduced in dimension, as we shall now describe.

## Model reduction

In this section, we discuss relevant dimensional parameter values, which motivate our choice of non-dimensionalisation (details are given in “Appendix”). The resulting dimensionless parameter values, and asymptotic expansion of the system variables in powers of the lumen aspect ratio, allow the system of governing equations and boundary conditions to be reduced significantly to four coupled PDEs. Imposing up- and down-stream boundary conditions closes the problem and eliminates two of these equations, leaving a system of two coupled PDEs for the cell volume fraction and solute concentration at leading order in the lumen aspect ratio. The remaining leading order unknowns can be determined via analytical expressions.

### Parameter values

Typical dimensional parameter values are taken from the literature or guided by our experimental collaborators where possible. We present these in Table 1 along with corresponding dimensionless parameters in Table 2. Dimensional parameter values for which data could not be found are omitted from Table 1; instead, their dimensionless equivalents are defined in Table 2 and values chosen so that our asymptotic analysis retains as many features as possible at leading order. A few choices are worth mentioning here: for a more in-depth discussion see Pearson et al. (2013). Firstly, we note that the aspect ratio of each region is small; in particular, we define the lumen aspect ratio by $\varepsilon = h_1/L \ll 1$ and use this as the relevant parameter for our asymptotic expansion in Sect. 3.2. In addition, the reduced Reynolds number in the lumen and upper fluid layer is $\varepsilon ^2\mathrm {Re} = 2.05\times 10^{-6}$ which justifies neglecting inertial effects in these regions in Sect. 2.1 and motivates our choice of non-dimensionalisation. We set the viscosity ratio $\mu _\mathrm {w}/\mu _\mathrm {n} = \lambda \varepsilon$ for some constant $\lambda = {O}(1)$. This is motivated by the fact that we would expect the effective (macroscale) viscosity of the cell phase to be much greater than that of water as a result of the cytoskeletal network, the cell–scaffold affinity and microscale cell–cell interactions. We choose the dimensionless cell–scaffold drag $\zeta _\mathrm {ns}$ and water–scaffold drag $\zeta _\mathrm {ws}$ to be of ${O}(1)$, so that their effects are retained in the leading order model, but assume that $\zeta _\mathrm {ws} < \zeta _\mathrm {ns}$ (as would be expected physically).

**Table 1:** Dimensional parameters

Parameter	Dimensional value and units	Definition
$h_1$	$200~\upmu$m$^{\mathrm {a}}$	Lumen height
$h_2$	$200~\upmu$m$^{\mathrm {a}}$	Porous membrane height
$h_3+h_4$	$600~\upmu$m$^{\mathrm {a}}$	ECS height (cell layer $+$ upper fluid layer height)
$L$	$0.1~$m$^{\mathrm {a}}$	Length of bioreactor modelling domain
$\rho _\mathrm {w}$	1 g cm$^{-3\ \mathrm {a}}$	Water density
$\mu _\mathrm {w}$	$10^{-3}$ Pa s$^{\mathrm {a}}$	Water viscosity
$k$	$6.73 \times 10^{-16}$ m$^{2\ \mathrm {b}}$	Porous membrane permeability
$p_{\mathrm{atm}}$	14.69 psia$^{\mathrm {a}}$	Atmospheric pressure
$Q_{\mathrm {l,in}}, Q_\mathrm {f,in}$	$1.02\times 10^{-11} - 1.02\times 10^{-8}$ m$^2$ s$^{-1\ \mathrm {c}}$	Lumen/upper fluid layer inlet flux
$\varGamma _{\mathrm {nw}}$	$5.79 \times 10^{-6}$ s$^{-1\ \mathrm {c}}$	cell proliferation rate coefficient
$\varGamma _{\mathrm {wn}}$	$4.13 \times 10^{-7}$ s$^{-1}$	Cell death rate coefficient
$c_{\mathrm{in}}$	$0.22$ mol m$^{-3\ \mathrm {a}}$	Inlet solute concentration
$D$	$3\times 10^{-9}$ m$^2$ s$^{-1\ \mathrm {a}}$	Solute diffusion coefficient in water
$U^*$	$1.0239 \times 10^{-8}$ m s$^{-1\ \mathrm {c}}$	Typical porous membrane velocity
$C^*$	$0.22$ mol m$^{-3\ \mathrm {a}}$	Typical solute concentration

$^{\mathrm {a}}$ Values taken from Shipley et al. (2010)

$^{\mathrm {b}}$ Experimentally obtained values

$^{\mathrm {c}}$ Values based on estimations by our experimental collaborators

**Table 2:** Dimensionless parameters

Parameter	Definition	Value	Restriction (if any)
$\hat{h}_2$	$h_2/(\varepsilon L)$	1	$\hat{h}_2 > 0$
$\hat{h}_3 + \hat{h}_4$	$(h_3+h_4)/(\varepsilon L)$	3	$\hat{h}_3, \hat{h}_4 > 0$
$\theta _\mathrm {s}$	Scaffold volume fraction	$0.4^{\mathrm {a}}$	$0<\theta _\mathrm {s}<1$
$\varepsilon$	$h_1/L$	$2\times 10^{-3}$	$0 < \varepsilon \ll 1$
$\lambda$	$\mu _\mathrm {w}/(\varepsilon \mu _\mathrm {n})$	1	$\varepsilon \ll \lambda \ll 1/\varepsilon$
$\varepsilon ^2$Pe	$\varepsilon LU^*/(\lambda D)$	$6.83\times 10^{-4}$	$\varepsilon ^3 \ll \varepsilon ^2\mathrm {Pe} \ll \varepsilon$
$\varepsilon ^2$Re	$\varepsilon \rho _\mathrm {w} LU^*/ (\lambda \mu _\mathrm {w})$	$2.05\times 10^{-6}$	$\varepsilon ^2 \mathrm {Re} \ll 1$
$\phi$	Porous membrane porosity	0.77$^{\mathrm {b}}$	$0 < \phi < 1$
$\kappa$	$k / (\lambda \varepsilon ^5 L^2)$	2.1	$\varepsilon \ll \kappa \ll 1/\varepsilon$
$\hat{Q}_{i,\mathrm {in}},\ i=\mathrm {l,f}$	$\lambda Q_{i,\mathrm {in}}/(L U^*)$	0.01–10	$\varepsilon \ll \hat{Q_{i,\mathrm {in}}} \ll 1/\varepsilon$
$\hat{\nu }$	$\lambda \varepsilon ^3 L \nu / (\mu _w U^*)$	0.3$^{{\mathrm {c}}}$	–
$\hat{\delta }$	$\lambda \varepsilon ^3 L \delta / (\mu _w U^*)$	0.1$^{{\mathrm {c}}}$	–
$\hat{\eta }$	$\lambda \varepsilon ^3 L \eta / (\mu _w U^*)$	0.3$^{{\mathrm {c}}}$	–
$\zeta _{\mathrm {ns}}$	$\gamma _{\mathrm {ns}}L^2\lambda \varepsilon ^3 / \mu _\mathrm {w}$	1	$\varepsilon \ll \zeta _{\mathrm {ns}} \ll 1/\varepsilon$
$\zeta _{\mathrm {ws}}$	$\gamma _{\mathrm {ws}}L^2\lambda \varepsilon ^3 / \mu _\mathrm {w}$	0.1	$\varepsilon \ll \zeta _{\mathrm {ws}} \ll \zeta _{\mathrm {ns}}$
$\hat{\varGamma }_{\mathrm {nw}}$	$L \varGamma _{\mathrm {nw}}/ U^*$	$56.52$	$\varepsilon \ll \hat{\varGamma }_{\mathrm {nw}} \ll 1/\varepsilon$
$\hat{\varGamma }_{\mathrm {wn}}$	$L \varGamma _{\mathrm {wn}}/ U^*$	$4.04$	$\varepsilon \ll \hat{\varGamma }_{\mathrm {wn}} \ll 1/\varepsilon$
$\hat{\varGamma }_{\mathrm {R}1}$	$L^2 \varGamma _{\mathrm {R}1} / (C^*D)$	50	$\varepsilon \ll \hat{\varGamma }_{\mathrm {R}1} \ll 1/\varepsilon$
$\hat{K}, \hat{K}_1$	$K/C^*, K_1/C^*$	1	–
$\hat{c}_{\mathrm {in}}$	$c_{\mathrm {in}} / C^*$	1	–
$P_\mathrm {d}$	–	2.5	–

Values taken from $^{\mathrm {a}}$Lemon et al. (2006), $^{\mathrm {b}}$Meneghello et al. (2009), and $^{{\mathrm {c}}}$O’Dea et al. (2010)

We define the velocity scale $U^*$ to be a typical horizontal velocity of the water in the porous membrane. We then take the corresponding horizontal velocity scale in the cell layer to be $U^*$, and in both the lumen and upper fluid layer to be $\mu _\mathrm {n}U^*/ \mu _\mathrm {w}$, so that the same pressure scales apply throughout the system in our non-dimensionalisation. These choices are also motivated by the fact that we would expect the porous structures to hinder the fluid flow in the membrane and cell layer. In addition, this choice enables substantial analytical progress to be made in the mathematical reduction in Sect. 3.2. We note that other limits could be considered, for instance taking the cell and water phases to be of comparable viscosity (as in O’Dea et al. 2010, 2013a) or the cell phase to be inviscid (as in Lemon et al. 2006).

We take the reduced Péclet number in the lumen and upper fluid layer, $\varepsilon ^2$Pe, to be order $\varepsilon ^2$ so that diffusion dominates advection throughout. The concentration scale $C^*$ and inlet concentration $c_\mathrm {in}$ are both set to be a typical oxygen concentration used in cell culture (Shipley and Waters 2012), as oxygen is the solute of interest in Sect. 4. The two-dimensional lumen and upper fluid inlet fluxes $Q_\mathrm {l,in}$ and $Q_\mathrm {f,in}$ (which come into the up- and down-stream boundary conditions in Sect. 3.3) have been converted from three-dimensional experimental values by first calculating the corresponding velocity and then multiplying by the length scale in the $y$ direction, $\varepsilon L$. The downstream lumen outlet pressure $P_\mathrm {d}$ and atmospheric pressure $p_\mathrm {atm}$ are also introduced in Sect. 3.3. The cell proliferation/death rates $\varGamma _\mathrm {nw}/\varGamma _\mathrm {wn}$ and solute uptake rate $\varGamma _{\mathrm {R}_1}$ are parameters in the constitutive laws for $J_\mathrm {n}$ and $\mathcal {R}$, both of which we define in Sect. 4. Based on estimations by our experimental collaborators, we take the (dimensional) cell proliferation rate coefficient $\varGamma _\mathrm {nw}$ to correspond to one cell division every 48 h, and we assume that cells live, on average, for 28 days when fixing the (dimensional) cell death rate coefficient $\varGamma _\mathrm {wn}$.

### Derivation of the reduced model

Having non-dimensionalised the governing equations and boundary conditions (see “Appendix”), we asymptotically expand each dependent variable in powers of $\varepsilon$, for instance setting $u_\mathrm {l} \sim u_{\mathrm {l}_0} + \varepsilon u_{\mathrm {l}_1} + \varepsilon ^2 u_{\mathrm {l}_2} + \cdots$ and similarly for the remaining velocities, reduced pressures, solute concentrations and volume fractions. In the following, we omit the subscript $0$ from leading order variables except where needed for clarity. Equating coefficients of $\varepsilon ^0$ in the lumen then yields

3.1a $$\begin{aligned}&\frac{\partial u_\mathrm {l}}{\partial x} + \frac{\partial v_\mathrm {l}}{\partial y} = 0,\end{aligned}$$

3.1b $$\begin{aligned}&\frac{\partial ^2 u_\mathrm {l}}{\partial y^2} = \frac{\partial p_\mathrm {l}}{\partial x},\end{aligned}$$

3.1c $$\begin{aligned}&\frac{\partial p_\mathrm {l}}{\partial y} = 0,\end{aligned}$$

3.1d $$\begin{aligned}&\frac{\partial ^2 c_\mathrm {l}}{\partial y^2} = 0; \end{aligned}$$

in the membrane

3.2a $$\begin{aligned}&u_\mathrm {m} \equiv 0,\end{aligned}$$

3.2b $$\begin{aligned}&v_\mathrm {m} = -\kappa \frac{\partial p_\mathrm {m}}{\partial y},\end{aligned}$$

3.2c $$\begin{aligned}&\frac{\partial ^2 p_\mathrm {m}}{\partial y^2} = 0,\end{aligned}$$

3.2d $$\begin{aligned}&\frac{\partial ^2 c_\mathrm {m}}{\partial y^2} = 0; \end{aligned}$$

in the cell layer

3.3a $$\begin{aligned}&\theta _\mathrm {n}+\theta _\mathrm {w}+\theta _\mathrm {s}=1,\end{aligned}$$

3.3b $$\begin{aligned}&\frac{\partial \theta _\mathrm {n}}{\partial t} + \frac{\partial }{\partial x}(\theta _\mathrm {n}u_\mathrm {n}) + \frac{\partial }{\partial y}(\theta _\mathrm {n}v_\mathrm {n}) = J_\mathrm {n},\end{aligned}$$

3.3c $$\begin{aligned}&\frac{\partial \theta _\mathrm {w}}{\partial t} + \frac{\partial }{\partial x}(\theta _\mathrm {w}u_\mathrm {w}) + \frac{\partial }{\partial y}(\theta _\mathrm {w}v_\mathrm {w}) = -J_\mathrm {n},\end{aligned}$$

3.3d $$\begin{aligned}&\quad -\,\theta _\mathrm {n}\frac{\partial p_\mathrm {w}}{\partial x}-\frac{\partial }{\partial x}(\theta _\mathrm {n}\varPi ) + \theta _\mathrm {s}\psi _\mathrm {ns}\frac{\partial \theta _\mathrm {n}}{\partial x} \nonumber \\&\quad -\,\zeta _\mathrm {ns}\theta _\mathrm {n}\theta _\mathrm {s}u_\mathrm {n} + \frac{\partial }{\partial y}\left( \theta _\mathrm {n}\frac{\partial u_\mathrm {n}}{\partial y}\right) = 0,\end{aligned}$$

3.3e $$\begin{aligned}&\quad -\,\theta _\mathrm {n}\frac{\partial p_\mathrm {w}}{\partial y} - \frac{\partial }{\partial y}(\theta _\mathrm {n}\varPi ) + \theta _\mathrm {s}\zeta _\mathrm {ns}\frac{\partial \theta _\mathrm {n}}{\partial y} = 0,\end{aligned}$$

3.3f $$\begin{aligned}&\theta _\mathrm {w}\frac{\partial p_\mathrm {w}}{\partial x}+\zeta _\mathrm {ws}\theta _\mathrm {w}\theta _\mathrm {s} u_\mathrm {w}= 0,\end{aligned}$$

3.3g $$\begin{aligned}&\theta _\mathrm {w}\frac{\partial p_\mathrm {w}}{\partial y} = 0,\end{aligned}$$

3.3h $$\begin{aligned}&\frac{\partial }{\partial y}\left( \theta _\mathrm {w}\frac{\partial c_\mathrm {w}}{\partial y}\right) = 0; \end{aligned}$$

and in the upper fluid layer

3.4a $$\begin{aligned}&\frac{\partial u_\mathrm {f}}{\partial x} + \frac{\partial v_\mathrm {f}}{\partial y} = 0,\end{aligned}$$

3.4b $$\begin{aligned}&\frac{\partial ^2 u_\mathrm {f}}{\partial y^2} = \frac{\partial p_\mathrm {f}}{\partial x},\end{aligned}$$

3.4c $$\begin{aligned}&\frac{\partial p_\mathrm {f}}{\partial y} = 0,\end{aligned}$$

3.4d $$\begin{aligned}&\frac{\partial ^2 c_\mathrm {f}}{\partial y^2} = 0. \end{aligned}$$

The leading order boundary conditions on the lumen centreline are

3.5a $$\begin{aligned}&\frac{\partial u_\mathrm {l}}{\partial y} = 0,\end{aligned}$$

3.5b $$\begin{aligned}&v_\mathrm {l} = 0,\end{aligned}$$

3.5c $$\begin{aligned}&\frac{\partial c_\mathrm {l}}{\partial y} = 0\quad \mathrm {on}\ y=0; \end{aligned}$$

on the lumen/membrane interface

3.6a $$\begin{aligned}&u_\mathrm {l} = v_\mathrm {l} = 0,\end{aligned}$$

3.6b $$\begin{aligned}&p_\mathrm {l} = p_\mathrm {m},\end{aligned}$$

3.6c $$\begin{aligned}&c_\mathrm {l} = c_\mathrm {m},\end{aligned}$$

3.6d $$\begin{aligned}&\frac{\partial c_\mathrm {l}}{\partial y} = \phi \frac{\partial c_\mathrm {m}}{\partial y}\quad \mathrm {on}\ y=1; \end{aligned}$$

on the membrane/cell layer interface

3.7a $$\begin{aligned}&u_\mathrm {n} = v_\mathrm {n} = 0,\end{aligned}$$

3.7b $$\begin{aligned}&v_\mathrm {w} = -\frac{\kappa \phi }{\theta _\mathrm {w}}\frac{\partial p_\mathrm {m}}{\partial y},\end{aligned}$$

3.7c $$\begin{aligned}&p_\mathrm {m} = p_\mathrm {w},\end{aligned}$$

3.7d $$\begin{aligned}&c_\mathrm {m} = c_\mathrm {w},\end{aligned}$$

3.7e $$\begin{aligned}&\phi \frac{\partial c_\mathrm {m}}{\partial y} = \theta _\mathrm {w}\frac{\partial c_\mathrm {w}}{\partial y}\quad \mathrm {on}\ y=1+h_2; \end{aligned}$$

on the cell layer/upper fluid interface

3.8a $$\begin{aligned}&v_\mathrm {n} = 0,\end{aligned}$$

3.8b $$\begin{aligned}&\frac{\partial u_\mathrm {n}}{\partial y} = 0,\end{aligned}$$

3.8c $$\begin{aligned}&u_\mathrm {f} = v_\mathrm {f} = 0,\end{aligned}$$

3.8d $$\begin{aligned}&p_\mathrm {w} = p_\mathrm {f},\end{aligned}$$

3.8e $$\begin{aligned}&c_\mathrm {w} = c_\mathrm {f},\end{aligned}$$

3.8f $$\begin{aligned}&\theta _\mathrm {w}\frac{\partial c_\mathrm {w}}{\partial y} = \frac{\partial c_\mathrm {f}}{\partial y}\quad \mathrm {on}\ y=1+h_2+h_3; \end{aligned}$$

and finally on the bioreactor top

3.9a $$\begin{aligned}&u_\mathrm {f} = 0,\end{aligned}$$

3.9b $$\begin{aligned}&v_\mathrm {f}= 0,\end{aligned}$$

3.9c $$\begin{aligned}&\frac{\partial c_\mathrm {f}}{\partial y} = 0\quad \mathrm {on}\ y=H. \end{aligned}$$

Consideration of the above solute equations and boundary conditions shows that at leading order, there is a global concentration that is independent of $y$: $c_\mathrm {l} = c_\mathrm {m} = c_\mathrm {w} = c_\mathrm {f} = c(x,t)$. To close the problem and find $c(x,t)$, we must also consider the solute equations at ${O}(\varepsilon ^2)$. We note that the ${O}(\varepsilon )$ components of the solute equations and boundary conditions in each layer indicate that $\partial c_{i_1}/\partial y = 0 (i=\mathrm {l},\mathrm {m},\mathrm {w},\mathrm {f})$, and so (in the lumen, membrane, cell layer and upper fluid layer, respectively)

3.10a $$\begin{aligned}&\mathrm {Pe}\left( \frac{\partial }{\partial x}(c_\mathrm {l}u_\mathrm {l}) + \frac{\partial }{\partial y}(c_\mathrm {l}v_\mathrm {l})\right) = \frac{\partial ^2 c_\mathrm {l}}{\partial x^2} + \frac{\partial ^2 c_{\mathrm {l},2}}{\partial y^2},\end{aligned}$$

3.10b $$\begin{aligned}&\frac{\partial ^2 c_\mathrm {m}}{\partial x^2} + \frac{\partial ^2 c_{\mathrm {m},2}}{\partial y^2} = 0,\end{aligned}$$

3.10c $$\begin{aligned}&\frac{\partial }{\partial x}\left( \theta _\mathrm {w}\frac{\partial c_\mathrm {w}}{\partial x}\right) + \frac{\partial }{\partial y}\left( \theta _\mathrm {w}\frac{\partial c_{\mathrm {w},2}}{\partial y} + \theta _{\mathrm {w}_2}\frac{\partial c_\mathrm {w}}{\partial y}\right) \nonumber \\&\qquad +\,\mathcal {R} = 0,\end{aligned}$$

3.10d $$\begin{aligned}&\mathrm {Pe}\left( \frac{\partial }{\partial x}(c_\mathrm {f}u_\mathrm {f}) + \frac{\partial }{\partial y}(c_\mathrm {f}v_\mathrm {f})\right) = \frac{\partial ^2 c_\mathrm {f}}{\partial x^2} + \frac{\partial ^2 c_{\mathrm {f},2}}{\partial y^2}, \end{aligned}$$

together with boundary conditions

3.11 $$\begin{aligned} \frac{\partial c_{\mathrm {l},2}}{\partial y} = 0\quad \mathrm {on}\ y=0, \end{aligned}$$

3.12a $$\begin{aligned}&\frac{\partial c_{\mathrm {l},2}}{\partial y} = \phi \frac{\partial c_{\mathrm {m},2}}{\partial y},\end{aligned}$$

3.12b $$\begin{aligned}&c_{\mathrm {l},2} = c_{\mathrm {m},2}\quad \mathrm {on}\ y=1, \end{aligned}$$

3.13a $$\begin{aligned}&\phi \frac{\partial c_{\mathrm {m},2}}{\partial y} = \theta _\mathrm {w}\frac{\partial c_{\mathrm {w},2}}{\partial y},\end{aligned}$$

3.13b $$\begin{aligned}&c_{\mathrm {m},2} = c_{\mathrm {w},2}\quad \mathrm {on}\ y=1+h_2, \end{aligned}$$

3.14a $$\begin{aligned}&\theta _\mathrm {w}\frac{\partial c_{\mathrm {w},2}}{\partial y} = \frac{\partial c_{\mathrm {f},2}}{\partial y},\end{aligned}$$

3.14b $$\begin{aligned}&c_{\mathrm {w},2} = c_{\mathrm {f},2}\quad \mathrm {on}\ y=1+h_2+h_3, \end{aligned}$$

3.15 $$\begin{aligned} \frac{\partial c_{\mathrm {f},2}}{\partial y} = 0\quad \mathrm {on}\ y=H. \end{aligned}$$

A substantial amount of analytical progress can be made in the distinguished limit considered above. Equations (3.1c), (3.3g) and (3.4c) respectively tell us that the leading order fluid pressures in the lumen, cell layer and upper fluid layer are independent of $y$. From (3.3e), and given $\theta _\mathrm {s}$ is constant, we can thus conclude that the cell volume fraction $\theta _\mathrm {n}$ is also a function of $x$ and $t$ only, which allows substantial simplifications to be made. We can obtain the following expressions for the leading order variables in terms of $p_\mathrm {l}(x,t), p_\mathrm {f}(x,t), \theta _\mathrm {n}(x,t)$ and $c(x,t)$:

3.16 $$\begin{aligned}&u_\mathrm {l} = \frac{1}{2}\frac{\partial p_\mathrm {l}}{\partial x}(y^2-1),\quad v_\mathrm {l}\equiv 0,\quad c_\mathrm {l} = c(x,t),\end{aligned}$$

3.17 $$\begin{aligned}&\nonumber u_\mathrm {m} \equiv 0,\quad v_\mathrm {m} = -\frac{\kappa }{h_2} (p_\mathrm {w}-p_\mathrm {l}),\\&p_\mathrm {m} = \frac{1}{h_2}(p_\mathrm {w}-p_\mathrm {l})(y-1) + p_\mathrm {l},\quad c_\mathrm {m} = c(x,t), \end{aligned}$$

3.18a $$\begin{aligned} u_\mathrm {w}&= -\frac{1}{\theta _\mathrm {s}\zeta _\mathrm {ws}}\frac{\partial p_\mathrm {w}}{\partial x},\end{aligned}$$

3.18b $$\begin{aligned} p_\mathrm {w}&= p_\mathrm {f}(x,t),\end{aligned}$$

3.18c $$\begin{aligned} c_\mathrm {w}&= c(x,t), \end{aligned}$$

3.19 $$\begin{aligned} u_\mathrm {n}&=\frac{M(x,t)}{\zeta _\mathrm {ns}\theta _\mathrm {s}}\left\{ \frac{ \cosh \left( \sqrt{\zeta _\mathrm {ns}\theta _\mathrm {s}}\left( 1+h_2+h_3- y\right) \right) }{\cosh \left( \sqrt{\zeta _\mathrm {ns}\theta _\mathrm {s}}h_3\right) } - 1\right\} ,\end{aligned}$$

3.20 $$\begin{aligned} \nonumber u_\mathrm {f}&= \frac{1}{2}\frac{\partial p_\mathrm {w}}{\partial x}\left( y^2 + (h_4-2H)(y-H)-H^2\right) ,\\ v_\mathrm {f}&\equiv 0,\quad c_\mathrm {f} = c(x,t), \end{aligned}$$

where $M(x,t)$ is given by

3.21 $$\begin{aligned}&M(x,t) = \frac{\partial p_\mathrm {w}}{\partial x} +\frac{1}{\theta _\mathrm {n}} \varPhi (\theta _\mathrm {n}) \frac{\partial \theta _\mathrm {n}}{\partial x}, \end{aligned}$$

3.22 $$\begin{aligned}&\Phi (\theta _\mathrm {n}) = \varPi + \theta _\mathrm {n}\varPi '_\mathrm {n} - \psi _\mathrm {ns}\theta _\mathrm {s}. \end{aligned}$$

Hence, at leading order, we have Poiseuille flow in the lumen and upper fluid layer and transverse flow only in the porous membrane. In the cell layer, the horizontal fluid flow is Darcy-like. The variables $v_\mathrm {n}$ and $v_\mathrm {w}$ can be determined from the leading order conservation of mass Eqs. (3.3b, c), respectively.

In addition, we have the following equations for $p_\mathrm {l}, \theta _\mathrm {n}$, the global leading order concentration $c$ and $p_\mathrm {f}$:

3.23a $$\begin{aligned}&\frac{\partial ^2 p_\mathrm {l}}{\partial x^2} = 0,\end{aligned}$$

3.23b $$\begin{aligned}&\frac{\partial \theta _\mathrm {n}}{\partial t} + \frac{\partial Q_\mathrm {n}}{\partial x} = J_\mathrm {n},\end{aligned}$$

3.23c $$\begin{aligned}&\frac{\partial Q_\mathrm {c}}{\partial x} = -h_3\mathcal {R},\end{aligned}$$

3.23d $$\begin{aligned}&\frac{\partial ^2 p_\mathrm {f}}{\partial x^2} = 0, \end{aligned}$$

where the cell flux $Q_\mathrm {n}$ is defined by

3.24 $$\begin{aligned} Q_\mathrm {n}&= \frac{M(x,t)}{\zeta _\mathrm {ns}\theta _\mathrm {s}} \left( \frac{\tanh (\alpha _3)}{\alpha _3}-1\right) \theta _\mathrm {n}, \quad \alpha _3 =\sqrt{\zeta _\mathrm {ns}\theta _\mathrm {s}}h_3, \end{aligned}$$

and the solute flux $Q_\mathrm {c}$ by

3.25 $$\begin{aligned} Q_\mathrm {c}&= \left( \frac{1}{3}\mathrm {Pe}\frac{\partial p_\mathrm {l}}{\partial x} + \frac{h_4^3}{3}\mathrm {Pe}\frac{\partial p_\mathrm {f}}{\partial x}\right) c \nonumber \\&\qquad + (1+\phi h_2 + \theta _\mathrm {w}h_3 + h_4)\frac{\partial c}{\partial x}. \end{aligned}$$

The equations for $p_\mathrm {l}, \theta _\mathrm {n}$ and $p_\mathrm {f}$ come from integrating the respective conservation of mass Eqs. (3.1a), (3.3b) and (3.4a) across the lumen, cell layer and upper fluid layer. The remaining equation (3.23c) has been obtained by adding together the ${O}(\varepsilon ^2)$ solute equations (3.10a–d) after averaging each across the appropriate region. We now impose up- and down-stream boundary conditions to close the problem.

### Boundary conditions for the reduced model

We mimic the experimental set-up where fluid is pumped into the bioreactor via both the lumen inlet and (upstream) ECS port, and so impose

3.26 $$\begin{aligned} Q_\mathrm {l,in}&= \int _{0}^{1}u_\mathrm {l}\, \mathrm {d}y\qquad \;\;\mathrm {at}\ x=0,\end{aligned}$$

3.27 $$\begin{aligned} Q_\mathrm {f,in}&= \int _{H-h_4}^{H}u_\mathrm {f}\, \mathrm {d}y\quad \mathrm {at}\ x=0, \end{aligned}$$

where $Q_\mathrm {l,in}$ and $Q_\mathrm {f,in}$ are prescribed (dimensionless) volume fluxes per unit length in the $z$ direction (perpendicular to $x$ and $y$) into the lumen and upper fluid layer, respectively, and are assumed constant. We note that in this case, the dimensional $Q_\mathrm {f,in}$ is of the same order as the dimensional $Q_\mathrm {l,in}$ given that the velocity scales in the lumen and upper fluid layer are the same. We also prescribe a down-stream lumen pressure and mimic the atmospheric pressure conditions at the down-stream ECS ports by setting

3.28 $$\begin{aligned} p_\mathrm {l}&= P_\mathrm {d},\quad p_\mathrm {f} = 0\quad \mathrm {at}\ x=1. \end{aligned}$$

For the cells, we impose no flux out of the modelling domain (which would be achieved using filters in an experiment):

3.29 $$\begin{aligned} Q_\mathrm {n}&= 0\quad \mathrm {at}\ x=0,1. \end{aligned}$$

Finally, we prescribe an inlet solute concentration and assume no diffusive flux at the downstream end of the bioreactor:

3.30 $$\begin{aligned}&c=1\quad \mathrm {at}\ x=0,\end{aligned}$$

3.31 $$\begin{aligned}&\frac{\partial c}{\partial x} = 0\quad \mathrm {at}\ x=1. \end{aligned}$$

As there is no downstream constraint on the solute concentration experimentally, condition (3.31) is motivated by the fact that we would expect the concentration to be constant in space as it leaves the bioreactor, due to the effects of diffusion.

Applying boundary conditions (3.26)–(3.28) allows us to solve (3.23a, d) explicitly for $p_\mathrm {l}$ and $p_\mathrm {f}$, giving

3.32 $$\begin{aligned} p_\mathrm {l} = 3Q_\mathrm {l,in}(1-x) + P_\mathrm {d},\quad p_\mathrm {f} = \frac{12Q_\mathrm {f,in}}{h_4^3}(1-x), \end{aligned}$$

and thereby specific expressions for $u_\mathrm {l}, v_\mathrm {m}, p_\mathrm {m}, u_\mathrm {w}, p_\mathrm {w}$ and $u_\mathrm {f}$ from (3.16)–(3.19). In particular, we note the form for the fluid pressure in the cell layer, which is equal to $p_\mathrm {f}$ (see 3.18b),

3.33 $$\begin{aligned} p_\mathrm {w} = \frac{12Q_\mathrm {f,in}}{h_4^3}(1-x). \end{aligned}$$

### Summary of reduced model

In summary, we have derived the following coupled system to solve for $\theta _\mathrm {n}$ and $c$:

3.34a $$\begin{aligned}&\frac{\partial \theta _\mathrm {n}}{\partial t} + \frac{\partial Q_\mathrm {n}}{\partial x} = J_\mathrm {n},\end{aligned}$$

3.34b $$\begin{aligned}&\frac{\partial Q_\mathrm {c}}{\partial x} = -h_3\mathcal {R}, \end{aligned}$$

where

3.35 $$\begin{aligned}&Q_\mathrm {n} = \frac{M(x,t)}{\zeta _\mathrm {ns}\theta _\mathrm {s}} \left( \frac{\tanh (\alpha _3)}{\alpha _3}- 1\right) \theta _\mathrm {n},\end{aligned}$$

3.36 $$\begin{aligned}&Q_\mathrm {c} = -\mathrm {Pe}\left( Q_\mathrm {l,in} + 4Q_\mathrm {f,in}\right) c + b(\theta _\mathrm {n})\frac{\partial c}{\partial x},\end{aligned}$$

3.37 $$\begin{aligned}&b(\theta _\mathrm {n}) = \left( 1+\phi h_2 + \theta _\mathrm {w}h_3 + h_4\right) , \end{aligned}$$

with the boundary conditions

3.38 $$\begin{aligned}&Q_\mathrm {n} = 0\quad \mathrm {at}\ x=0,1,\end{aligned}$$

3.39 $$\begin{aligned}&c=1\quad \mathrm {at}\ x=0,\end{aligned}$$

3.40 $$\begin{aligned}&\frac{\partial c}{\partial x} = 0\quad \mathrm {at}\ x=1. \end{aligned}$$

Here $M$ and $\varPhi$ are as in (3.21) and (3.22), and the appropriate initial condition is the prescription of $\theta _\mathrm {n}$ at $t=0$ for $0<x<1$. We can compare this to the corresponding system from Pearson et al. (2013): it is interesting to see that, despite the apparently more complex set-up here (with four modelling regions instead of three), we can reduce the governing equations to a system of two instead of three coupled PDEs. This is due to the addition of the upper fluid layer region which was not present in Pearson et al. (2013), and in which we can explicitly solve for the reduced water pressure. The reduced water pressure in the cell layer is the same as that in the upper fluid layer, and hence only two unknowns remain, $\theta _\mathrm {n}$ and $c$.

## Numerical results

The reduced system (3.34)–(3.40) was solved numerically using the method of lines, first discretising in $x$ and then performing the time integration using the MATLAB function ode15s. We are interested in steady states of the system, as these arise on timescales comparable to the long culture time of experiments (see Pearson et al. 2013 for a more thorough discussion). These states were found to be independent of the choice of initial condition in our simulations, but for completeness, we note that in the simulations presented in this paper, we set $\theta _\mathrm {n}(x,0)=0.3, c(x,0)=1$ for $0<x<1$.

We consider the effect of the fluid shear stress on the cell distribution and yield, in the case where the solute of interest is a nutrient (e.g. oxygen), and with the aim of determining optimal experimental conditions which result in both a spatially uniform cell population and high cell yield.

In each of the following sections, we consider the Michaelis–Menten type reaction term used in Pearson et al. (2013) for nutrient-limited proliferation:

4.1 $$\begin{aligned} \mathcal {R} = -\frac{\varGamma _{\mathrm {R}1}\theta _\mathrm {n}\theta _\mathrm {w}c}{K_1+c}. \end{aligned}$$

We take the cell mass transfer term $J_\mathrm {n}$ to have the form

4.2 $$\begin{aligned} J_\mathrm {n} = \frac{F(S)\varGamma _\mathrm {nw}\theta _\mathrm {n}\theta _\mathrm {w}c}{K+c} - \varGamma _\mathrm {wn}\theta _\mathrm {n}, \end{aligned}$$

where $S$ is the (dimensionless) fluid shear stress in the cell layer. If $F(S)$ were equal to unity, then we would recover the form used in the case of the nutrient-driven proliferation discussed in Pearson et al. (2013). In the following analysis, we take $F(S)$ to have different forms depending on the cells’ response to shear. As we effectively have Darcy flow of water through the cell layer, we use the following estimate for $S$ motivated by a similar expression in Whittaker et al. (2009):

4.3 $$\begin{aligned} S = \frac{\left| \frac{\partial p_\mathrm {w}}{\partial x}\right| }{\theta _\mathrm {w}}, \end{aligned}$$

based upon the assumption that there is Poiseuille flow through each of the ‘pores’ in the cell layer, where the pores are approximated as circular ducts. Given the assumed form for $S$, and the expression for $\partial p_\mathrm {w}/\partial x$ in (3.33), we can see that the fluid shear stress is dependent on both the upper fluid layer flux $Q_\mathrm {f,in}$ and cell layer width $h_3$ (via $h_4 = H-(1+h_2+h_3)$), both of which can be controlled experimentally. In what follows we investigate the sensitivity of our results to variations in these two parameters. Moreover, for a given flow rate and cell layer width, we note that variation in $S$ arises purely through variations in $\theta _\mathrm {w}$.

In each of the following case studies, we present results for the steady-state cell volume fraction $\theta _\mathrm {n}^*(x)$ and use the mean $\mu$ and standard deviation $\sigma$ of the cell volume fraction $\theta _\mathrm {n}$ to characterise the cell yield and distribution, where

4.4 $$\begin{aligned} \mu&= \int _{0}^{1}\theta _\mathrm {n}^*(x)\, \mathrm {d}x,\end{aligned}$$

4.5 $$\begin{aligned} \sigma&= \sqrt{\int _{0}^{1}\left( \theta _\mathrm {n}^*(x) - \mu \right) ^2\, \mathrm {d}x}. \end{aligned}$$

### Shear stress-enhanced proliferation

First of all, we consider the case when cell proliferation is enhanced by increased levels of fluid shear stress. This has been observed in cells such as osteoblasts (Kapur et al. 2003), chondrocytes (Malaviya and Nerem 2002) and (more controversially) endothelial progenitor cells (Yamamoto et al. 2003), and is thought to be an important factor in ensuring the mechanical integrity of tissue such as bone and cartilage (Bancroft et al. 2002; Gemmiti and Guldberg 2006). The exact nature of this dependence is not known and will depend on the cell type in question. To capture the qualitative aspects of this dependence, we set

**Fig. 2:**
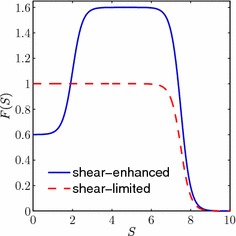
Plot of $F(S)$, showing the dependence of cell proliferation on the fluid shear stress in the case where increasing shear stress first enhances cell proliferation (*solid line*, Sect. 4.1), and where increasing shear stress inhibits cell proliferation (*dashed line*, Sect. 4.2)

4.6 $$\begin{aligned} F(S) = 0.3 + 0.5\tanh (2S-4) - 0.8\tanh (2S-15),\quad \quad \end{aligned}$$

so that the cell proliferation rate increases with shear stress up to a maximal level, but (as would be expected) high levels of shear stress result in a reduced proliferation rate (see Fig. 2). The coefficients in (4.6) have been chosen to capture the possible behaviours of the cell population within the range of computed shear stresses in our model.

**Fig. 3:**
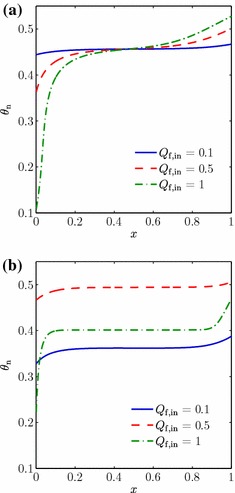
Plots of $\theta _\mathrm {n}$ for a range of values of the upper fluid layer flux $Q_\mathrm {f,in}$, in the **a** shear-insensitive and **b** shear-sensitive (enhanced proliferation) cases. The lumen flux $Q_\mathrm {l,in}=0.1$, the cell layer width $h_3=1$ and other parameter values are as in Table 2

Results from this case study can be seen in Fig. 3, where the steady-state cell population distribution obtained with $F(S)$ as in Eq. (4.6) is compared to that when $F(S)=1$ (denoted the shear-insensitive population, i.e. when the fluid shear has no effect on the cells) for a range of upper fluid layer flow rates $Q_\mathrm {f,in}$. From Fig. 3a, b, we see that for the lowest flow rate of $Q_\mathrm {f,in}=0.1$, the cell volume fraction of the shear-sensitive (hereby called shear-enhanced) population is less than that of the shear-insensitive population for $0<x<1$, but then increases above the shear-insensitive volume fraction once $Q_\mathrm {f,in}$ is increased to 0.5. This corresponds to the shear stress exceeding the value at which $F(S)$ is increased above 1 (around 2.5) and is to be expected given our chosen form of $F(S)$. The increase in $\theta _\mathrm {n}$ is observed across the whole bioreactor domain, instead of at a specific point, as a result of the relatively small variation in $\theta _\mathrm {n}$ (and hence $\theta _\mathrm {w}$) for $0<x<1$, which in turn means that there is little spatial variation in $S$. At the intermediate flow rate ($Q_\mathrm {f,in}=0.5$), we also see that the shear-enhanced population becomes much more spatially uniform than the shear-insensitive cells. For the highest flow rate $Q_\mathrm {f,in}=1$, the shear-enhanced $\theta _\mathrm {n}$ decreases at each point in $x$ as the shear stress reaches levels which decrease the cell proliferation rate. In addition, greater up- and down-stream variation is seen in $\theta _\mathrm {n}$ for both populations, but the changes are less extreme for the shear-enhanced cells. Hence, overall, the effect of the shear stress is to ‘smooth out’ the cell population: an increase in $\theta _\mathrm {n}$ corresponds to a decrease in $\theta _\mathrm {w}$, and thus a higher value of $S$. Thus, regions where $\theta _\mathrm {n}$ is relatively high experience a fluid shear stress sufficient to decrease $F(S)$ and hence the proliferation rate, whilst regions where $\theta _\mathrm {n}$ is relatively low experience a lower shear stress and therefore increased proliferation rate.

**Fig. 4:**
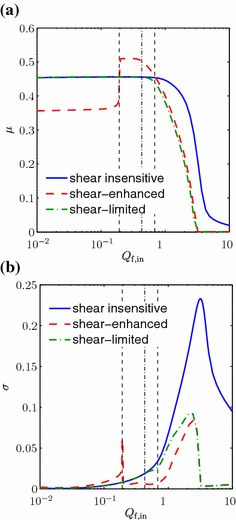
Plots of (**a**) the mean $\mu$ and (**b**) the standard deviation $\sigma$ of $\theta _\mathrm {n}$, for a range of $Q_\mathrm {f,in}$ values in the shear-insensitive, shear-enhanced proliferation and shear-limited proliferation cases. The *vertical dashed lines* indicate the optimal flux range for the shear-enhanced case, within which the cell yield is higher than the shear-insensitive population. The *vertical dash-dotted line* indicates the critical flux value for the shear-limited case, below which the shear-limited and shear-insensitive cases are indistinguishable. The lumen flux $Q_\mathrm {l,in}=0.1$, the cell layer width $h_3=1$ and other parameter values are as in Table 2

The effect of shear on the cell yield and population uniformity can be seen in Fig. 4, and the plots support the findings discussed above. The mean $\mu$ and standard deviation $\sigma$ of $\theta _\mathrm {n}$ are plotted for a range of values of $Q_\mathrm {f,in}$, for the shear-enhanced and shear-insensitive cases. This clearly shows that the shear-enhanced population has a lower yield and greater standard deviation than the shear-insensitive population for low $Q_\mathrm {f,in}$ values, but that this relationship is reversed when $Q_\mathrm {f,in}$ increases beyond a critical value just below $0.2$. This switch corresponds to the critical value of $S$ being reached at which $F(S) > 1$, and the cell proliferation rate is enhanced. As can be seen in the graphs for both $\mu$ and $\sigma$, the change occurs relatively abruptly; this is due to the fact that (as mentioned above) there is relatively little variation in $\theta _\mathrm {n}$ at this value of $Q_\mathrm {f,in}$, and so the fluid shear stress is relatively constant along the domain. The standard deviation of the shear-enhanced population remains lower than the shear-insensitive population for all subsequent values of $Q_\mathrm {f,in}$, but the yield drops below that of the shear-insensitive population once another critical value of $Q_\mathrm {f,in}$ is reached, around 0.7, when the value of $F(S)$ falls below $1$. Thus, between these critical values (given by vertical dashed lines in Fig. 4), there is an optimum range of $Q_\mathrm {f,in}$ within which shear stress helps to promote cell yield and also population uniformity. Within this range, the most uniform population is obtained at the (optimal) flow rate of $Q_\mathrm {f,opt}=0.2048$. We also note that, for a flow rate above the first critical value, the shear-enhanced population is always more spatially uniform than the shear-insensitive population for a given yield.

### Shear stress-limited proliferation

In the second case study, we take $F(S)$ to be of the following form

4.7 $$\begin{aligned} F(S) = \frac{1}{2}\left( 1 - \tanh (2S-15)\right) , \end{aligned}$$

so that cell proliferation is inhibited for sufficiently high values of fluid shear stress (see Fig. 2). Cells that have been shown to be sensitive to shear stress in this way include smooth muscle cells (Papadaki et al. 1996) and certain types of endothelial cells (e.g. human aortic endothelial cells Imberti et al. 2002 and human umbilical vein endothelial cells Akimoto et al. 2000). As in the previous section, the exact form of the cells’ shear stress dependence is unknown, and our choice of $F(S)$ and corresponding parameter values have been chosen so that our model captures the range of possible behaviours. Throughout this section, the cell population with proliferation rate limited by shear stress will be known as shear-limited.

**Fig. 5:**
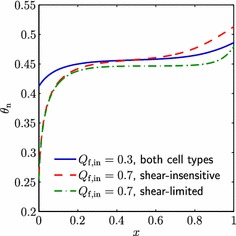
Plots of $\theta _\mathrm {n}$ in the shear-sensitive (limited proliferation) and shear-insensitive cases, for a range of values of the upper fluid layer flux $Q_\mathrm {f,in}$. The lumen flux $Q_\mathrm {l,in}=0.1$, the cell layer width $h_3=1$ and other parameter values are as in Table 2

Figure 5 shows the results obtained from this case study. For low values of $Q_\mathrm {f,in}$, the shear stress has no effect on cell proliferation (as $F(S)$ is equal to $1$) and thus the shear-limited and shear-insensitive population distributions are the same. As $Q_\mathrm {f,in}$ is increased, however, the downstream region, where $\theta _\mathrm {n}$ is greater, experiences a higher level of shear stress, and thus a decrease in proliferation rate of the shear-limited population. This results in the shear-limited population becoming more uniform than the shear-insensitive population. In contrast to the previous section, we note that regions of lower cell volume fraction are unchanged by the shear sensitivity, since in this case, proliferation is never enhanced by the fluid shear. Thus, the increased uniformity of the shear-limited population is at a cost of a lower overall volume fraction. The plots of $\mu$ and $\sigma$ in Fig. 4 show that there is just one critical value of $Q_\mathrm {f,in}$ in this case (around 0.4, given by the vertical dash-dotted line in Fig. 4), below which the shear-limited and shear-insensitive cases are indistinguishable. Above this, the shear-limited population is more uniform than the shear-insensitive population, but also has a lower yield. However, Table 3 demonstrates that the percentage decrease in $\sigma$ is more significant than the percentage decrease in $\mu$ for $0.4 \lesssim Q_\mathrm {f,in} \lesssim 0.8$, and thus, this provides a potentially optimal flow rate range when a uniform cell population is more important than a high cell yield.

**Table 3:** Comparison of cell yield $\mu$ and standard deviation $\sigma$ for the shear-limited and shear-insensitive cases, for a range of $Q_\mathrm {f,in}$ values

$Q_\mathrm {f,in}$	Stress–insensitive	Stress–limited	% Change
$$\mu $$			
0.4	0.4557	0.4557	$-5\times 10^{-4}$
0.6	0.4548	0.4493	$-1.209$
0.8	0.4523	0.422	$-6.695$
1	0.4464	0.3827	$-14.28$
$$\sigma $$			
0.4	0.0174	0.0174	$-0.013$
0.6	0.0279	0.0234	$-16.06$
0.8	0.0438	0.0343	$-21.67$
1	0.0671	0.053	$-21.0$

### Sensitivity to cell layer height

**Fig. 6:**
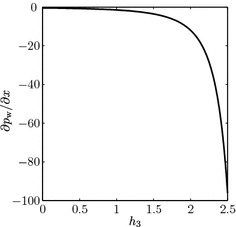
Plot of $\partial p_\mathrm {w}/\partial x$ versus the cell layer width $h_3$, for fixed $Q_\mathrm {f,in}=~1$

**Fig. 7:**
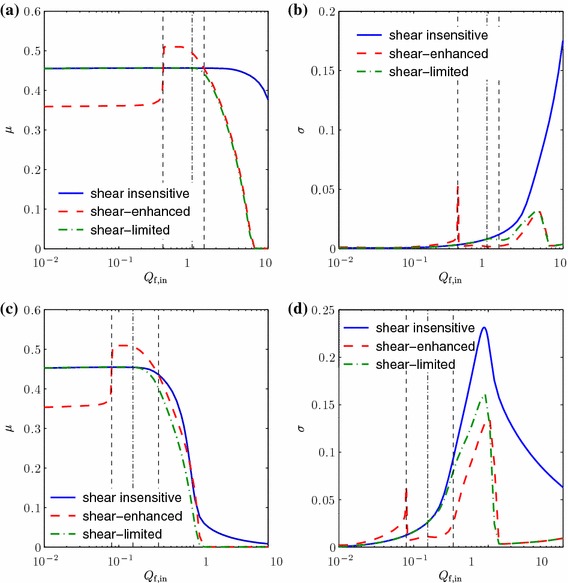
Plots of the mean $\mu$ and standard deviation $\sigma$ of $\theta _\mathrm {n}$, for a range of $Q_\mathrm {f,in}$ values in the shear-insensitive, shear-enhanced proliferation and shear-limited proliferation cases, for (**a, b**) $h_3=0.5$ and (**c, d**) $h_3=1.5$. The *vertical dashed lines* again indicate the optimal flux range for the shear-enhanced case, and the *vertical dash-dotted line* the critical flux value for the shear-limited case. The lumen flux $Q_\mathrm {l,in}=0.1$ and other parameter values are as in Table 2

Finally, we investigate the sensitivity of our results to the cell layer height $h_3$, which may be prescribed experimentally. In each case, we compare the cell volume fraction $\theta _\mathrm {n}$ and note that larger values of $h_3$ correspond to greater overall cell numbers, and vice versa for smaller values of $h_3$. The cell layer height also affects the pressure gradient in the cell and upper fluid layers, since $\partial p_\mathrm {w}/\partial x$ depends linearly on $h_4^{-1} = \left( H - (1+h_2+h_3)\right) ^{-1}$ (see Fig. 6). Figure 7a, b shows plots of the mean $\mu$ and standard deviation $\sigma$ of $\theta _\mathrm {n}$ for a range of values of $Q_\mathrm {f,in}$ for a thinner cell layer than Fig. 4, whilst Fig. 7c, d shows the corresponding plots for a thicker cell layer. In Fig. 7a, we see that the optimal ranges for $Q_\mathrm {f,in}$ are now higher. The corresponding optimal flow rate is also higher than before, with $Q_\mathrm {f,opt}=1.048$. This is a result of the form for $\left| \partial p_\mathrm {w}/\partial x\right|$, which increases as $h_3$ does as stated above. The shear stress therefore also decreases as $h_3$ decreases, and we thus need higher flow rates in order to obtain the same behaviour from the cell population. As expected, this trend is reversed in Fig. 7c when $h_3$ takes a higher value, with an optimal flow rate in this case of $Q_\mathrm {f,opt}=0.085$. In addition, for $Q_\mathrm {f,in}$ greater than around $1$, we see the cell population decreases significantly, no matter what the shear stress dependence. We therefore conclude that a thinner cell layer makes the cell population less sensitive to the upper fluid layer flow rate, as higher flow rates can be used before compromising cell yield, and vice versa for a thicker cell layer.

## Discussion

We have developed a multiphase model of fluid flow, solute transport and cell distribution in a simplified HFMB. This extends the work presented by Pearson et al. (2013), by considering the alternative set-up in which the cell–scaffold construct only partially occupies the ECS and there is an additional upper layer of free-flowing fluid. Fluid is pumped into the upper fluid layer via the upstream ECS port, and hence, this region experiences higher flow rates than when fluid is only supplied at the lumen inlet. It is therefore hypothesised that this could result in a greater fluid shear stress in the cell layer which could have an important, and possibly beneficial, effect on cell dynamics. This is confirmed from our model results which imply that the fluid shear stress in the cell layer is dependent on both the upper fluid inlet flux and cell layer width, both of which are varied as part of our investigation.

We presented results from two different case studies. In the first (relevant to osteoblasts, chondrocytes or endothelial progenitor cells), cell proliferation rate was enhanced by fluid shear stress, but only up to a certain critical level. Results demonstrated that the mechanosensitive cell population required a certain minimum ECS flow rate in order to reach yields comparable to the shear-insensitive population. For flow rates that resulted in fluid shear within the ‘enhancing’ range, however, the mechanosensitive population had a higher yield and was more uniform than the shear-insensitive cells. This uniformity persisted even once the shear levels became detrimental to the proliferation rate and the yield decreased. In the second case study, fluid shear had no effect on cell proliferation until a critical value was surpassed, after which it decreased the proliferation rate. This could be applicable to smooth muscle cells, or certain types of endothelial cells. Results again showed that the mechanosensitive cell population was more uniform than the shear-insensitive cells, but always had a lower cell yield. However, an ideal flow rate range could again be found, within which the mechanosensitive population is more uniform than the shear-insensitive population, and the yield has not yet been significantly compromised. Finally, we considered the effect of the cell layer width on these results and found that a thinner cell layer experiences a lower level of shear stress than a thicker layer, and hence can withstand higher flow rates.

The results presented here are qualitative in nature, demonstrating the range of expected behaviours in each situation. In particular, the functional forms for $F(S)$ are constitutive and relatively simple, having been chosen to clearly demonstrate the possible effects of mechanotransduction on cell distribution in a HFMB. Any choice of $F(S)$ would need experimental validation to be applied to a specific experimental setup. There are currently no relevant experimental results to which we can compare the results from our mathematical model, as the modelling is ahead of the experiments. However, the model framework developed here is extremely flexible and permits interrogation of different forms for any of the parameter values and constitutive laws presented. Hence, once relevant data become available, the model could be readily adapted and applied to a particular cell population, and proper validation would be possible. This could then be used in a predictive way to stimulate future experimental work.

## Appendix: Non-dimensionalisation

Motivated by the discussion in Sect. 3.1, we non-dimensionalise as follows:

6.1 $$\begin{aligned}&x = L\hat{x},\quad y = \varepsilon L\hat{y},\quad t = \frac{L}{U^*}\hat{t},\nonumber \\&h_i = \varepsilon L \hat{h}_i\quad (i = 2,3,4),\quad H = \varepsilon L \hat{H},\nonumber \\&u_i = \frac{\mu _\mathrm {n}}{\mu _\mathrm {w}}U^{*}\hat{u}_i,\quad v_i = \frac{\varepsilon \mu _\mathrm {n}}{\mu _\mathrm {w}}U^{*}\hat{v}_i\quad (i=\mathrm {l},\mathrm {f}),\nonumber \\&u_i = U^*\hat{u}_i,\quad v_i = \varepsilon U^*\hat{v}_i\quad (i=\mathrm {m},\mathrm {w},\mathrm {n}),\nonumber \\&p_i = p_{\mathrm {atm}} + \frac{\mu _\mathrm {n} U^*}{\varepsilon ^2 L}\hat{p}_i\quad (i=\mathrm {l},\mathrm {m},\mathrm {w},\mathrm {n},\mathrm {f}),\nonumber \\&\varPi = \frac{\mu _\mathrm {n} U^*}{\varepsilon ^2 L}\hat{\varPi },\quad \psi _{\mathrm {ns}} = \frac{\mu _\mathrm {n} U^*}{\varepsilon ^2 L}\hat{\psi }_{\mathrm {ns}},\nonumber \\&c_i = C^*\hat{c}_i\quad (i=\mathrm {l},\mathrm {m},\mathrm {w},\mathrm {f}),\nonumber \\&\mathcal {R} = \frac{DC^*}{L^2}\hat{\mathcal {R}},\quad J_\mathrm {n} = \frac{U^{*}}{L}\hat{J}_\mathrm {n}, \end{aligned}$$

where $C^*$ is a typical concentration scale, and we have employed a viscous pressure scaling throughout (motivated by lubrication theory as stated above). In choosing the timescale $L/U^*$ and mass transfer scale $U^*/L$ for $J_\mathrm {n}$, we have assumed that the timescales for advection in the porous membrane/cell layer and proliferation are comparable.

The dimensionless governing equations in the lumen are therefore (dropping hats on dimensionless variables)

6.2 $$\begin{aligned}&\frac{\partial u_\mathrm {l}}{\partial x} + \frac{\partial v_\mathrm {l}}{\partial y} = 0,\quad -\frac{\partial p_\mathrm {l}}{\partial x} + \varepsilon ^2\frac{\partial ^2 u_\mathrm {l}}{\partial x^2} + \frac{\partial ^2 u_\mathrm {l}}{\partial y^2} = 0,\nonumber \\&\quad -\frac{\partial p_\mathrm {l}}{\partial y} + \varepsilon ^4\frac{\partial ^2 v_\mathrm {l}}{\partial x^2} + \varepsilon ^2\frac{\partial ^2 v_\mathrm {l}}{\partial y^2} =0,\nonumber \\&\quad \varepsilon ^2\mathrm {Pe}\left( \lambda \varepsilon \frac{\partial c_\mathrm {l}}{\partial t} + \nabla \cdot (c_\mathrm {l}\mathbf {u}_\mathrm {l})\right) = \varepsilon ^2\frac{\partial ^2 c_\mathrm {l}}{\partial x^2} + \frac{\partial ^2 c_\mathrm {l}}{\partial y^2}; \end{aligned}$$

and in the porous membrane (dividing through by $\phi$ in the nutrient transport equation)

6.3 $$\begin{aligned}&u_\mathrm {m} = -\varepsilon ^2 \kappa \frac{\partial p_\mathrm {m}}{\partial x},\quad v_\mathrm {m} = -\kappa \frac{\partial p_\mathrm {m}}{\partial y},\nonumber \\&\quad \varepsilon ^2 \frac{\partial ^2 p_\mathrm {m}}{\partial x^2} + \frac{\partial ^2 p_\mathrm {m}}{\partial y^2} = 0,\nonumber \\&\quad \lambda \varepsilon ^3\mathrm {Pe}\left( \frac{\partial c_\mathrm {m}}{\partial t} + \nabla \cdot (c_\mathrm {m}\mathbf {u}_\mathrm {m})\right) = \varepsilon ^2\frac{\partial ^2 c_\mathrm {m}}{\partial x^2} + \frac{\partial ^2 c_\mathrm {m}}{\partial y^2}, \end{aligned}$$

where $\kappa = k/(\lambda \varepsilon ^5 L^2)$ is the ${O}(1)$ dimensionless permeability (see Table 2). In the cell layer the no-voids condition is

6.4 $$\begin{aligned} \theta _\mathrm {n} + \theta _\mathrm {w} + \theta _\mathrm {s} = 1; \end{aligned}$$

the cell equations are

6.5 $$\begin{aligned}&\frac{\partial \theta _\mathrm {n}}{\partial t} + \frac{\partial }{\partial x} (\theta _\mathrm {n}u_\mathrm {n}) + \frac{\partial }{\partial y}(\theta _\mathrm {n} v_\mathrm {n}) = J_\mathrm {n},\nonumber \\&\quad -\,\theta _\mathrm {n}\frac{\partial p_\mathrm {w}}{\partial x} - \frac{\partial }{\partial x}(\theta _\mathrm {n}\varPi ) + \psi _{\mathrm {ns}}\theta _\mathrm {s}\frac{\partial \theta _\mathrm {n}}{\partial x}\nonumber \\&\quad +\,\frac{2\varepsilon ^2}{3}\frac{\partial }{\partial x}\left( \theta _\mathrm {n} \left( 2\frac{\partial u_\mathrm {n}}{\partial x} - \frac{\partial v_\mathrm {n}}{\partial y}\right) \right) \nonumber \\&\quad +\,\frac{\partial }{\partial y}\left( \theta _\mathrm {n} \left( \frac{\partial u_\mathrm {n}}{\partial y} + \varepsilon ^2 \frac{\partial v_\mathrm {n}}{\partial x}\right) \right) - \zeta _{\mathrm {ns}}\theta _\mathrm {n}\theta _\mathrm {s}u_\mathrm {n} = 0,\nonumber \\&\quad -\,\theta _\mathrm {n}\frac{\partial p_\mathrm {w}}{\partial y} - \frac{\partial }{\partial y}(\theta _\mathrm {n}\varPi ) + \psi _{\mathrm {ns}} \theta _\mathrm {s}\frac{\partial \theta _\mathrm {n}}{\partial y}\nonumber \\&\quad +\,\varepsilon ^2\frac{\partial }{\partial x}\left( \theta _\mathrm {n} \left( \frac{\partial u_\mathrm {n}}{\partial y} + \varepsilon ^2\frac{\partial v_\mathrm {n}}{\partial x}\right) \right) \nonumber \\&\quad +\,\frac{2\varepsilon ^2}{3}\frac{\partial }{\partial y} \left( \theta _\mathrm {n}\left( 2\frac{\partial v_\mathrm {n}}{\partial y} - \frac{\partial u_\mathrm {n}}{\partial x}\right) \right) - \varepsilon ^2\zeta _{\mathrm {ns}}\theta _\mathrm {n}\theta _\mathrm {s}v_\mathrm {n} = 0; \nonumber \\ \end{aligned}$$

the water equations are

6.6 $$\begin{aligned}&\frac{\partial \theta _\mathrm {w}}{\partial t} + \frac{\partial }{\partial x}(\theta _\mathrm {w}u_\mathrm {w}) + \frac{\partial }{\partial y}(\theta _\mathrm {w}v_\mathrm {w}) = -J_\mathrm {n},\nonumber \\&\quad -\,\theta _\mathrm {w}\frac{\partial p_\mathrm {w}}{\partial x} - \zeta _{\mathrm {ws}}\theta _\mathrm {w}\theta _\mathrm {s}u_\mathrm {w} = 0,\nonumber \\&\quad -\,\theta _\mathrm {w}\frac{\partial p_\mathrm {w}}{\partial y} - \varepsilon ^2\zeta _{\mathrm {ws}}\theta _\mathrm {w}\theta _\mathrm {s}v_\mathrm {w} = 0; \end{aligned}$$

and the solute equation is

6.7 $$\begin{aligned}&\lambda \varepsilon ^3\mathrm {Pe}\left( \frac{\partial }{\partial t}(\theta _\mathrm {w}c_\mathrm {w}) + \nabla \cdot (\theta _\mathrm {w}c_\mathrm {w}\mathbf {u}_\mathrm {w})\right) \nonumber \\&\quad = \varepsilon ^2\frac{\partial }{\partial x}\left( \theta _\mathrm {w}\frac{\partial c_\mathrm {w}}{\partial x}\right) +\frac{\partial }{\partial y}\left( \theta _\mathrm {w}\frac{\partial c_\mathrm {w}}{\partial y}\right) + \varepsilon ^2\mathcal {R}, \end{aligned}$$

where $\zeta _{ij} = \gamma _{ij}L^2\varepsilon ^2/\mu _\mathrm {n}$ for $i=\mathrm {n},\mathrm {w},j=\mathrm {s}$ are dimensionless. In the upper fluid layer

6.8 $$\begin{aligned}&\frac{\partial u_\mathrm {f}}{\partial x} + \frac{\partial v_\mathrm {f}}{\partial y} = 0,\quad -\frac{\partial p_\mathrm {f}}{\partial x} + \varepsilon ^2\frac{\partial ^2 u_\mathrm {f}}{\partial x^2} + \frac{\partial ^2 u_\mathrm {f}}{\partial y^2} = 0,\nonumber \\&\quad -\frac{\partial p_\mathrm {f}}{\partial y} + \varepsilon ^4\frac{\partial ^2 v_\mathrm {f}}{\partial x^2} + \varepsilon ^2\frac{\partial ^2 v_\mathrm {f}}{\partial y^2} =0,\nonumber \\&\quad \varepsilon ^2\mathrm {Pe}\left( \lambda \varepsilon \frac{\partial c_\mathrm {f}}{\partial t} + \nabla \cdot (c_\mathrm {f}\mathbf {u}_\mathrm {f})\right) = \varepsilon ^2\frac{\partial ^2 c_\mathrm {f}}{\partial x^2} + \frac{\partial ^2 c_\mathrm {f}}{\partial y^2}. \end{aligned}$$

The dimensionless boundary conditions on the lumen centreline are

6.9 $$\begin{aligned} \frac{\partial u_\mathrm {l}}{\partial y}&= 0,\quad v_\mathrm {l} = 0,\quad \frac{\partial c_\mathrm {l}}{\partial y}=0\quad \mathrm {on}\quad y = 0; \end{aligned}$$

on the lumen/membrane interface (substituting in for $p_\mathrm {m}$ in place of $v_\mathrm {m}$)

6.10 $$\begin{aligned}&u_\mathrm {l} = 0,\quad \varepsilon \frac{\partial p_\mathrm {m}}{\partial y} = -\frac{1}{\lambda \kappa \phi } v_\mathrm {l}, \nonumber \\&p_\mathrm {l} + \frac{2\varepsilon ^2}{3}\left( \frac{\partial u_\mathrm {l}}{\partial x} - 2\frac{\partial v_\mathrm {l}}{\partial y}\right) = p_\mathrm {m},\\&c_\mathrm {l}=c_\mathrm {m},\quad \frac{\partial c_\mathrm {l}}{\partial y} = \phi \frac{\partial c_\mathrm {m}}{\partial y}\quad \mathrm {on}\quad y = 1;\nonumber \end{aligned}$$

on the membrane/cell layer interface (again substituting in for $p_\mathrm {m}$ in place of $v_\mathrm {m}$)

6.11 $$\begin{aligned} \begin{aligned} u_\mathrm {n}&= v_\mathrm {n} = 0,\quad v_\mathrm {w} = -\frac{\kappa \phi }{\theta _\mathrm {w}} \frac{\partial p_\mathrm {m}}{\partial y},\quad p_\mathrm {w}=p_\mathrm {m},\\ c_\mathrm {m}&= c_\mathrm {w},\quad \phi \frac{\partial c_\mathrm {m}}{\partial y} = \theta _\mathrm {w} \frac{\partial c_\mathrm {w}}{\partial y}\quad \mathrm {on}\quad y = 1 + h_2; \end{aligned} \end{aligned}$$

on the cell/upper fluid layer interface

6.12 $$\begin{aligned}&v_\mathrm {n} = 0,\quad \frac{\partial u_\mathrm {n}}{\partial y} + \varepsilon ^2\frac{\partial v_\mathrm {n}}{\partial x} = 0,\quad u_\mathrm {f} = 0,\nonumber \\&\lambda \varepsilon \theta _\mathrm {w}v_\mathrm {w} = v_\mathrm {f},\quad -p_\mathrm {w} = -p_\mathrm {f} + 2\varepsilon ^2\frac{\partial v_\mathrm {f}}{\partial y},\\&c_\mathrm {w} = c_\mathrm {f},\quad \theta _\mathrm {w}\frac{\partial c_\mathrm {w}}{\partial y} = \frac{\partial c_\mathrm {f}}{\partial y}\quad \mathrm {on}\quad y=1+h_2+h_3;\nonumber \end{aligned}$$

and finally on the bioreactor top

6.13 $$\begin{aligned} u_\mathrm {f}&= v_\mathrm {f} = 0,\quad \frac{\partial c_\mathrm {f}}{\partial y}=0\quad \mathrm {on}\quad y = H. \end{aligned}$$
